# *CECR1* p.Gly47Arg mutations are not increased in frequency in Turkish Behçet's disease patients compared with healthy controls

**DOI:** 10.1186/1546-0096-13-S1-P64

**Published:** 2015-09-28

**Authors:** B Erer, E Remmers, M Takeuchi, D Ustek, I Tugal-Tutkun, E Seyahi, Y Ozyazgan, A Gul, M Ombrello, D Kastner

**Affiliations:** 1 Istanbul University Istanbul Medical Faculty, Internal Medicine Division of Rheumatology, Istanbul, Turkey; 2 National Institutes of Health, National Human Genome Research Institute, Bethesda, MD, USA; 3 Istanbul University Institute of Experimental Medicine, Istanbul, Turkey; 4 Istanbul University Istanbul Medical Faculty, Istanbul, Turkey; 5 Istanbul University Cerrahpasa Medical Faculty, Istanbul, Turkey; 6 National Institutes of Health, National Institutes of Arthritis Musculoskeletal and Skin Diseases, Bethesda, MD, USA

## Question

In Behçet's disease (BD), vasculitis involving blood vessels of nearly all sizes and types may underlie the diverse tissue and organ involvement. Loss of function mutations in the *CECR1* gene (encoding adenosine deaminase 2) have recently been shown to cause a recessive genetic disease, deficiency of adenine deaminase 2 (DADA2). Patients with DADA2 exhibit systemic vasculopathy characterized by intermittent fevers, skin rash, and neurovascular manifestations along with other features that can lead to a diagnosis of polyarteritis nodosa. Patients homozygous for the *CECR1* p.Gly47Arg mutation are reported in two non-consanguineous Turkish families and this mutation is found at low frequency in the Turkish population. We therefore attempted to determine whether some BD cases may be explained by adenosine deaminase 2 deficiency and whether this mutation contributes to BD risk in patients of Turkish ancestry.

## Methods

Turkish BD patients (n = 1,609) and controls (n = 1,519) were genotyped for p.Gly47Arg mutations in the *CECR1* gene using a Sequenom assay. The assay interrogated two mutant alleles of the first nucleotide of the Gly47 codon that both encode the glycine to arginine missense change.

## Results

We found p.Gly47Arg mutations in 4 BD patients and 3 healthy controls. No individuals (neither cases or controls) carried two mutant alleles. The carrier frequency for p.Gly47Arg mutations was 0.002 in cases and in controls.

## Conclusions

These data show that the carrier rate of *CECR1* p.Gly47Arg mutations is very low in Turkish Behcet's patients and not different from controls, suggesting no contribution to Behcet's disease.

